# 

**Published:** 1923-08

**Authors:** 


### HOSPITAL and HEALTH

REVIEW

*Established 1886* · *No.* 1865. *Vol. LXXII.*

*New Series.* Vol. II No. 23 *August* 1923 Price Sixpence

CONTENTS.

| | PAGE |
|---|---|
| Curing and Teaching | 289 |
| The Nemesis of Folly | 290 |
| Notes and Comments :— The Cottage Hospital—The Plus and the Minus of Sunshine—The Minister's Survey—Hospitals and Infirmaries—A Censorship of Modern Remedies—Controlling Infectious Disease in Canada—Lady Astor's Bill—Legacies to Hospitals—Radium and Life Changes—The Leper—Care of Pre-Tuberculous Infants in Paris—Auditing Bodies in Chicago—A Ten Years' Test Wanted—Hospital Wards and Private Patients—Consolidating Health Insurance Law—Health and Noise. | 291-2-3-4 |
| Zoning the Worker | 295 |
| The Prince of Wales at Guy's Hospital | 296 |
| The Voluntary Hospitals Report | 297 |
| The Public Health: Interviews with Local Authorities—Yarmouth | 299 |
| Hospital Accounts and Finance. By Joseph E. Stone, F.S.A.A. | 301 |
| The League of Remembrance | 302 |
| Pity the Poor Panel Patient | 303 |
| How a Country Hospital was Saved. By Walter Deacon | 304 |
| A Separate-Pavilion Hospital | 306 |
| The Horrors of Sanitation | 306 |
| Hospital Progress and Finance | 307 |
| Reviews of Books | 309 |
| Health Insurance Notes | 313 |
| Correspondence | 314 |
| The Royal Northern Hospital | 314 |
| The League of Mercy | 314 |
| Echoes of the Month | 315 |
| Hospital and Health News | 317 |
| Points from Hospital Reports | 319 |
| Public Health in Parliament | 320 |
| Recent Appointments | 320 |

